# Genetic background modulates phenotypes of serotonin transporter Ala56 knock-in mice

**DOI:** 10.1186/2040-2392-4-35

**Published:** 2013-10-01

**Authors:** Travis M Kerr, Christopher L Muller, Mahfuzur Miah, Christopher S Jetter, Rita Pfeiffer, Charisma Shah, Nicole Baganz, George M Anderson, Jacqueline N Crawley, James S Sutcliffe, Randy D Blakely, Jeremy Veenstra-VanderWeele

**Affiliations:** 1 Department of Psychiatry, Vanderbilt University, 465 21st Ave S, Nashville, TN 37232, USA; 2 Vanderbilt Brain Institute, Vanderbilt University, 465 21st Ave S, Nashville, TN 37232, USA; 3 Department of Pharmacology, Vanderbilt University, 465 21st Ave S, Nashville, TN 37232, USA; 4 Child Study Center, Yale University School of Medicine, 230 S Frontage Rd, New Haven, CT 06520, USA; 5 Department of Psychiatry and Behavioral Sciences, Medical Investigation of Neurodevelopmental Disorders (MIND) Institute, University of California Davis, 2825 50th St, Sacramento, CA 95817, USA; 6 Department of Molecular Physiology and Biophysics, 465 21st Ave S, Nashville, TN 37232, USA; 7 Silvio O. Conte Center for Neuroscience Research, 465 21st Ave S, Nashville, TN 37232, USA; 8 Department of Pediatrics, Vanderbilt University, 465 21st Ave S, Nashville, TN 37232, USA; 9 Treatment and Research Institute for Autism Spectrum Disorder, Vanderbilt Kennedy Center for Research on Human Development, 465 21st Ave S, Nashville, TN 37232, USA

**Keywords:** Serotonin, Monoamine, Transporter, Receptor, Autism, Compulsive

## Abstract

**Background:**

Previously, we identified multiple, rare serotonin (5-HT) transporter (SERT) variants in children with autism spectrum disorder (ASD). Although in our study the SERT Ala56 variant was over-transmitted to ASD probands, it was also seen in some unaffected individuals, suggesting that associated ASD risk is influenced by the epistatic effects of other genetic variation. Subsequently, we established that mice expressing the SERT Ala56 variant on a 129S6/S4 genetic background display multiple biochemical, physiological and behavioral changes, including hyperserotonemia, altered 5-HT receptor sensitivity, and altered social, communication, and repetitive behavior. Here we explore the effects of genetic background on SERT Ala56 knock-in phenotypes.

**Methods:**

To explore the effects of genetic background, we backcrossed SERT Ala56 mice on the 129 background into a C57BL/6 (B6) background to achieve congenic B6 SERT Ala56 mice, and assessed autism-relevant behavior, including sociability, ultrasonic vocalizations, and repetitive behavior in the home cage, as well as serotonergic phenotypes, including whole blood serotonin levels and serotonin receptor sensitivity.

**Results:**

One consistent phenotype between the two strains was performance in the tube test for dominance, where mutant mice displayed a greater tendency to withdraw from a social encounter in a narrow tube as compared to wildtype littermate controls. On the B6 background, mutant pup ultrasonic vocalizations were significantly increased, in contrast to decreased vocalizations seen previously on the 129 background. Several phenotypes seen on the 129 background were reduced or absent when the mutation was placed on the B6 background, including hyperserotonemia, 5-HT receptor hypersensivity, and repetitive behavior.

**Conclusions:**

Our findings provide a cogent example of how epistatic interactions can modulate the impact of functional genetic variation and suggest that some aspects of social behavior may be especially sensitive to changes in SERT function. Finally, these results provide a platform for the identification of genes that may modulate the risk of ASD in humans.

## Background

The serotonin (5-hydroxytryptamine, 5-HT) system has long been implicated in Autism Spectrum Disorder (ASD) [1]. Elevated whole blood 5-HT, termed hyperserotonemia, is found in about 30% of ASD patients [2,3]. Blood 5-HT levels are more heritable than ASD itself [4-7], but the link between hyperserotonemia and ASD pathophysiology has remained elusive. In the blood, 5-HT is found almost exclusively in platelets that cannot produce 5-HT, but take it up via the antidepressant-sensitive 5-HT transporter (SERT). Peripheral serotonin is synthesized and released by enterochromaffin cells that release 5-HT into the enteric circulation. Consistent with a primary role in platelet 5-HT uptake, polymorphisms in the SERT gene (*SLC6A4*) have been associated with human whole blood 5-HT levels; however, this has been observed only in males and not females [8], with a similar pattern observed for the SERT-interacting protein integrin β3 [8-10]. Several linkage studies have implicated the chromosome 17q region containing *SLC6A4* in ASD, also with stronger evidence in males [11-15].

In contrast to linkage studies, multiple groups have found, at most, modest association between common *SLC6A4* variants and ASD [16-18]. Allelic heterogeneity may account for difficulty in assessing association in this region. Indeed, multiple common functional variants have been identified in the *SLC6A4* gene (reviewed in [19]). We identified multiple, rare SERT coding variants in ASD families with evidence of linkage to 17q. We also found that the most common of these variants, SERT Ala56, was significantly associated with rigid-compulsive behavior and sensory aversion. Lending credence to a functional contribution of the SERT variants identified, each of the variants was found to confer increased 5-HT transport activity in cell models relative to wildtype SERT [14,20,21]. In our study, the Ala56 variant showed an approximately 2:1 transmission bias to affected versus unaffected children [14]. Importantly, however, some variant carriers were unaffected by ASD, and a case–control study has reported no association of ASD with rare SERT amino acid variants in the absence of linkage [22], indicating that other genetic or non-genetic risk factors likely impact the resulting phenotype.

To assess the functional relevance of the SERT Ala56 variant *in vivo*, we produced knock-in mice, expressing the variant on a 129S6/S4 inbred background. In these mice, we observed multiple biochemical and behavioral phenotypes that recapitulate some features of ASD, including hyperserotonemia, altered social function in the tube test and three-chamber sociability test, decreased ultrasonic vocalizations, and repetitive climbing/hanging behavior in the home cage [23]. We also observed hypersensitivity to *in vivo* activation of 5-HT_1A_ and 5-HT_2A_ receptors, reasonably attributable to diminished synaptic 5-HT availability and functional upregulation of 5-HT receptors. Unfortunately, this inbred strain background shows a low level of activity in novel environments across multiple behavioral tests [23], which limits the assessment of anxiety-like and social behaviors that are dependent upon spontaneous locomotion.

As 5-HT is a modulator of multiple CNS pathways subserving complex behaviors, we suspected that SERT coding variation could be influenced by genetic variation in other genes, which might account for the variable phenotype observed in humans with the SERT Ala56 allele. To explore this issue, we moved the SERT Ala56 allele to a C57BL/6 (B6) background for two purposes: 1) to examine autism-relevant behavior in a more active and social strain than 129, and 2) to study strain dependence of SERT function, the 5-HT system, and resulting behavior. Previous work identified a functional and behavioral impact of variation at two amino acids that differ between 129 and B6 inbred strains [24]. We therefore also backcrossed the 129 SERT onto the B6 background to allow a comparison with the mutant SERT on this background. Our findings reveal a striking influence of genetic background on the impact of the SERT Ala56 variant, suggesting that further dissection of strain differences could illuminate other genes modulating risk of ASD.

## Methods

### Mice

All animal procedures were in accordance with the National Institutes of Health Guide for the Care and Use of Laboratory Animals and were approved by the Vanderbilt University Institutional Animal Care and Use Committee.

### Backcross

Male SERT Ala56 mice (Glu39/Ala56/Arg152) on a mixed 129S6/SvEvTac and 129S4/ImJ background [23] were crossed with B6 females, with male progeny from each generation crossed again to B6 females for at least eight generations. The Jackson Lab Genome Scanning Service (Bar Harbor, ME, USA) was used to verify congenic status with B6. In parallel, wildtype 129S6/S4 mice from the same colony (SERT Glu39/Gly56/Arg152) were backcrossed at least ten generations to B6 and verified congenic with B6 to serve as valid control animals. SERT Gly56Ala and SERT Arg152/Lys genotyping was performed as described previously [23,24]. Sanger sequencing, as described previously, was used to verify that the SERT Ala56 was maintained after the full backcross [25].

### Behavioral testing

Co-housed littermate progeny of heterozygous pairs (SERT Gly56/Ala56) were used for all behavioral testing. A cohort of 20 matched male Ala56/Ala56 and Gly56/Gly56 littermates, as well as 16 co-housed matched male Ala56/Gly56 littermates, was used in behavioral experiments in the following order to minimize order effects on testing [26,27]: elevated zero maze, light–dark test, open field activity, three-chamber sociability test, tube test, Y maze spontaneous alternation, and von Frey filament test. A subset of this cohort was used for home cage monitoring (12 Ala56/Ala56 and 12 Gly56/Gly56). A cohort of 20 matched female Ala56/Ala56, Ala56/Gly56, and Gly56/Gly56 littermates was used in behavioral experiments in the following order: elevated zero maze, light–dark test, and open field activity.

### Elevated zero maze

Assessment of anxiety-like behaviors were evaluated using an elevated zero maze, as described previously [28] for 5 minutes under standard fluorescent light.

### Light–dark exploration

Anxiety-like responses were evaluated using activity monitors (Med Associates, Inc., St. Albans, VT, USA) divided in half to create a light compartment and a dark compartment, as described previously [29], with the subject mouse being placed in the light compartment at the beginning of the test and activity recorded for 5 minutes.

### Open field activity

Exploratory locomotor activity was evaluated in activity monitors (Med Associates, Inc., St. Albans, VT, USA), as described previously [23].

### Three-chamber sociability and preference for social novelty test

Social behavior was evaluated in a three-chamber polycarbonate apparatus with 4-inch sliding gates separating the 7 × 9-inch chambers, as described previously [23,30]. The subject mouse was initially allowed to explore all three chambers for 10 minutes to acclimate to the apparatus. A stimulus mouse (social stimulus) was then introduced inside an inverted wire pencil cup (Spectrum Diversified Designs, Streetsboro, OH, USA) in one side chamber with a clean empty pencil cup (inanimate stimulus) introduced in the opposite side chamber. The stimulus mouse was an adult male 129S4/ImJ mouse, previously habituated to the pencil cup in six 30-minute sessions across 3 days. The subject mouse was then allowed to explore all three chambers for 10 minutes. A new stimulus mouse was then introduced inside a pencil cup in the previous inanimate stimulus chamber. The subject mouse was finally allowed to explore all three chambers for 10 minutes. A research assistant blinded to mouse genotype coded videos for time spent in each chamber and within 1 cm of each pencil cup.

### Tube test

The tube test was conducted in male mice as described previously [23]. The apparatus is a 30-cm-long, 3.5-cm-diameter clear acrylic tube with small acrylic funnels added to each end to facilitate entry into the tube. Mice were preconditioned to the tube to reduce novelty effects and ensure that they will readily enter and progress forward through the tube. On two separate days before testing, each mouse was exposed to the tube, with progress through the tube resulting in the mouse being returned to the home cage. Mice that did not initially enter the tube were encouraged to run forward with a gentle pull of the tail. For each bout, a mouse was paired with a stranger mouse from a different cage. The 'winner’ mouse was declared when the other mouse backed out of the tube. Trials were repeated from the opposite end to avoid a side bias. Each mouse was tested against four to five individuals from other cages.

### Home cage monitoring

To evaluate possible repetitive behavior, 12 Ala56/Ala56 and 12 Gly56/Gly56 mice were video-recorded alone in their home cage for 24 hours while maintaining their light/dark schedule, as described previously [23]. Automated video analysis was conducted by using HomeCageScan (CleverSys, Inc., Reston, VA, USA) to index time spent performing individual behaviors. The resulting data were condensed into 10 individual behaviors: awaken/sleep, chew/eat/drink, rear, groom, hang, remain low, sniff, stretch, twitch, and walk. To normalize distributions for analysis, data were log_10_-transformed. Bouts of hanging behavior were defined as distinct periods of hanging from the wire lid of the cage separated by non-hanging behaviors. The number of bouts per animal was also log_10_-transformed for analysis.

### Y-maze

To evaluate for perseverative exploratory behavior, mice were tested for spontaneous alternation in the Y-maze, with maze arms of 6 × 35.5 cm, as previously described [31]. The subject mouse was placed facing the center in one of the three arms of the maze and then allowed to explore for 6 minutes. Each trial was video recorded and then scored for spontaneous alternation, which was defined as entry into a different arm than on the previous two entries.

### von Frey

Tactile sensitivity was assessed using plastic von Frey monofilaments applied to plantar surface of the subject’s right hind paw [32]. Briefly, each subject was placed into a plexiglass chamber with a wire mesh flooring for experimental access to the mouse footpad. Subjects were allowed to habituate to the testing chamber for 5 minutes before the assessment of tactile sensitivity. Von Frey monofilaments (Stoelting Co., Wood Dale, IL, USA) of ascending diameter were applied to the subject’s hind paw until reaching the target force (that is, bending of the filament). The lowest force required to elicit a paw withdrawal response was considered a subject’s tactile sensitivity threshold.

### Resident intruder

A subset of 18 male mice of each homozygous genotype was used, with two littermate cages removed due to previous fighting injuries. The test mouse, the resident, was isolated in its home cage for 7 days. A novel mouse, the intruder, was then introduced to the resident’s home cage for 5 minutes. The intruder was an adult male 129S4/ImJ mouse. Interactions were recorded and a researcher blind to genotype scored videos for time of the initial aggressive behavior of the resident.

### Ultrasonic vocalization

Progeny of heterozygous SERT Ala56/Gly56 pairs at postnatal day 7 were used to measure ultrasonic vocalizations, as described previously [23]. Pups were removed from their cage and placed in a Styrofoam chamber with bedding. Ultrasonic vocalizations were measured for 5 minutes using a Condenser ultrasound microphone (Avisoft-Bioacoustics, Berlin, Germany) and Avisoft SASLab Pro software (Avisoft-Bioacoustics, Berlin, Germany). Thresholds were set to detect only small frequency-modulated vocalizations within a 250-kHz range lasting at least 5 ms and occurring at least 20 ms apart.

### Dimethoxy-4-iodoamphetamine-induced head twitch response

A separate cohort of 12 littermate pairs was used to evaluate head twitch response to 2,5-dimethoxy-4-iodoamphetamine (DOI), as described previously [23]. Briefly, each mouse was administered an intraperitoneal injection of 1.0 mg/kg DOI (Sigma-Aldrich, St. Louis, MO, USA) and then allowed to acclimate to a standard housing cage with clean bedding for 34 minutes. Two research assistants who were blind to genotype then independently counted head twitches over 15 minutes.

### 8-hydroxy-2-(di-n-propylamino)tetralin-induced hypothermia

A separate cohort of 10 littermate pairs was used to evaluate hypothermia response to 8-hydroxy-2-(di-n-propylamino)tetralin (8-OH-DPAT). Briefly, core temperature was measured every 10 minutes for 80 minutes with a rectal probe (no. 50314; Stoelting, Wood Dale, IL, USA) connected to a BAT-12 thermometer (Physitemp Instruments, Clifton, NJ, USA). Immediately after the third temperature measurement, mice were administered a subcutaneous injection of 0.1 mg/kg 8-OH-DPAT (Sigma-Aldrich, St. Louis, MO, USA).

### Whole blood and midbrain harvest

Twelve SERT Ala56 mice and matched littermate controls at postnatal day 7 were rapidly decapitated and trunk blood was collected in 1.5 ml microcentrifuge tubes containing 37 USP units of lithium heparin and mixed by inversion. Samples were immediately placed on dry ice and then stored at -80°C until analysis.

### High-performance liquid chromatography

High-performance liquid chromatography (HPLC) was conducted as described previously [33]. Briefly, an internal standard solution (ISS) containing N-methylserotonin, ascorbic acid and sodium metabisulfite was added to the samples. After samples were vortex mixed, perchloric acid was added to the samples, which were mixed and then kept on ice for 10 minutes prior to centrifugation at 6000 g for 5 minutes. The supernatant was removed and stored at -80°C until analyzed by HPLC with fluorometric detection. Serotonin was determined with intra- and inter-assay coefficients of variation of less than 5 and 10%, respectively.

### Synaptosome 5-HT Uptake

Synaptosomes were prepared as described previously [34]. Briefly, 5-HT uptake was determined at 20 nM and 100 nM with tritiated 5-HT (Perkin Elmer, Waltham, MA, USA). Serotonin uptake was conducted at 37**°**C for five minutes, with SERT-specific uptake determined by subtracting S-citalopram blocked non-specific uptake. Resulting uptake data were normalized to total protein.

### Statistics

The primary analysis for each comparison was SERT Gly56/Gly56 versus SERT Ala56/Ala56. Paired t-tests were used for littermate pair comparisons, except where noted. For experiments that included heterozygous animals, secondary analysis with one-way ANOVA was used to evaluate overall effects of genotype, with post-hoc Bonferroni testing used only when the main effect or interaction was significant. For the three-chamber sociability test, repeated-measures ANOVA, excluding the center chamber, was used to evaluate social versus novel object for each genotype. All statistical analyses were performed on GraphPad Prism (La Jolla, CA, USA) or on the accompanying website.

## Results

As our previous work identified two sites of functional, coding variation in SERT between 129 and B6 inbred strains (Glu39Gly; Arg152Lys) [24], it was necessary to backcross 129-derived animals expressing wildtype Gly56 SERT in parallel with engineered 129 animals expressing SERT Ala56 onto the B6 background to permit a proper analysis of the unique effects of the Ala56 variant. This effort yielded animals with >99% C57BL/6 strain identity. A female C57BL/6 SERT ER-Gly56 mouse (SERT Glu39/Gly56/Arg152, hereafter described as Gly56) was bred with a male C57BL/6 SERT ER-Ala56 mouse (SERT Glu39/Ala56/Arg152, hereafter described as Ala56) to establish animals heterozygous for the SERT Ala56 variant, permitting the generation of homozygous Gly56/Gly56, heterozygous Gly56/Ala56, and homozygous Ala56/Ala56 littermates used in the experiments reported.

**Figure 1:**
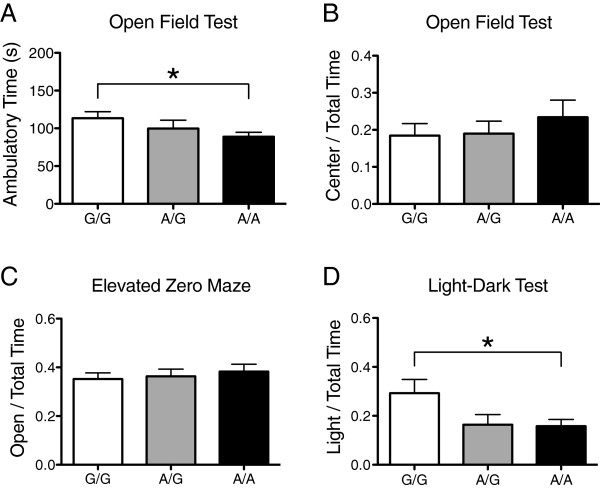
**Activity and anxiety-like behavior in male B6 SERT Ala56 knock-in mice. A)** Time (mean, standard error of the mean) spent ambulating during the first 5 minutes in the open field for wildtype littermate controls (G/G, n = 20), heterozygous (A/G, n = 16), and homozygous (A/A, n = 20) SERT Ala56 mice; **B)** Time spent in the center (>1 cm away from the side) of the open field divided by total time in the open field; **C)** Time spent in the open zone divided by total time in the elevated zero maze; **D)** Time spent in the light chamber divided by total time in the light–dark test. *P <0.05.

Whereas we found the 129-expressed Ala56 variant to induce no changes in locomotor activity in a novel environment, as assessed in the open field test [23], we detected a small but significant decrease in activity in homozygous male Ala56 animals on the B6 background, both for total ambulatory time (*t* = 3.45*, P* = 0.003) and ambulatory distance (*t* = 3.11*, P* = 0.006) (Figure 1A, [see Figure S1 in Additional file 1]). In contrast, females exhibited no genotype effects in open field activity [See Figure S2 in Additional file 2; See Figure S3 in Additional file 3].

Results for anxiety-like behavior conflicted across three tests. The ratio of time spent in the center of the open field, compared to total time, over the first five minutes of the test was not different in males (*t* = 0.97, *P* = 0.35) or females (*t* = 0.79*, P* = 0.44) (Figure 1B, [See Figure S2 in Additional file 2]). Similarly, no difference was seen in the time spent in the open arms of the Elevated Zero Maze over 5 minutes for males (*t* = 0.79*, P* = 0.44) or females (*t* = 1.52*, P* = 0.15) (Figure 1C, [See Figure S2 in Additional file 2]). In contrast, a genotype effect was observed with SERT Ala56 males spending less time in the light compartment of the light–dark test than homozygous Gly56 littermates (*t* = 2.51, *P* = 0.02) (Figure 1D). Again, females displayed no difference in this test (*t* = 1.55*, P* = 0.15) [See Figure S2 in Additional file 2].

**Figure 2:**
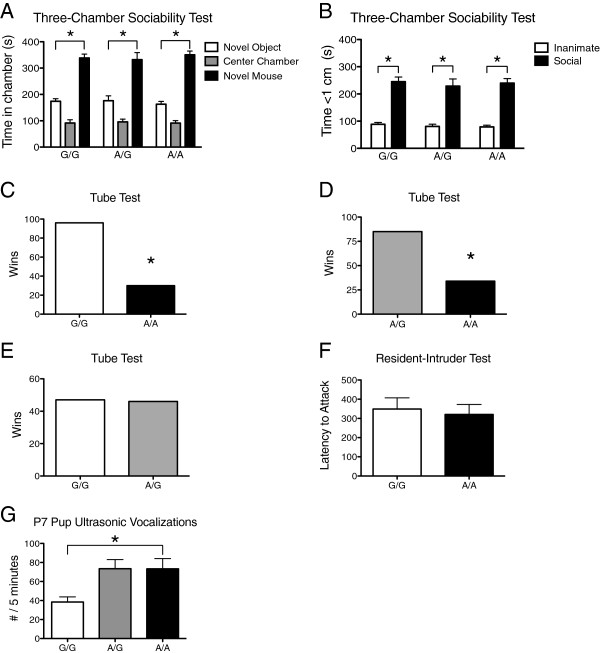
**Social behaviors and pup ultrasonic vocalizations in male B6 SERT Ala56 knock-in mice. A)** Time (mean, standard error of the mean) spent in the social stimulus chamber (novel mouse), center chamber, or inanimate stimulus chamber (novel object) in the three-chamber sociability test for wildtype littermate controls (G/G, n = 20), heterozygous (A/G, n = 16), and homozygous (A/A, n = 20) SERT Ala56 mice; **B)** Time spent within 1 cm of the social stimulus or inanimate stimulus in the three-chamber sociability test; **C)** Total number of wins (frontward exit) for pairings of wildtype littermate controls (G/G) and homozygous SERT Ala56/Ala56 (A/A) mice in the tube test confrontation; **D)** Total number of wins for heterozygous (A/G) versus homozygous SERT Ala56 (A/A) mice when paired in the tube test; **E)** Total number of wins for wildtype (G/G) versus heterozygous SERT Ala56 (A/G) mice when paired in the tube test; **F)** Latency to attack a wildtype 129S4 intruder mouse introduced into the cage of a resident subject mouse who had been singly housed for 7 days (n = 16 per genotype); **G)** Pup vocalizations upon separation from the dam for 5 min at postnatal day 7 for wildtype littermate controls (G/G, n = 36), heterozygous (A/G, n = 65), and homozygous SERT Ala56 (A/A, n = 33) mouse pups. *P <0.05.

Results on social testing were also mixed. In the three-chamber sociability test, all three genotypes showed a significant preference for the social stimulus over the non-social stimulus, both when chamber time was recorded (overall stimulus *F* = 96.6*, P* <0.0001 for each genotype) and when time <1 cm from the stimulus mouse was recorded (overall stimulus *F* = 135.1, *P* <0.0001 for each genotype) (Figure 2A,B). On the preference for social novelty portion of the three-chamber test, the Gly56 animal showed no significant preference for the novel social stimulus [See Figure S1 in Additional file 1], which makes the comparison data uninterpretable. On the tube test, homozygous Ala56 animals showed a significant propensity to back out of the tube when encountering a homozygous Gly56 animal (McNemar’s exact test *P* <0.0001; n = 126 encounters), as well as when encountering a Ala56/Gly56 animal (*P* <0.0001; n = 119 encounters) (Figure 2C,D). No difference was detected when heterozygous Ala56/Gly56 encountered Gly56/Gly56 animals (*P* = 1.0, n = 93 encounters) (Figure 2E). On the resident-intruder test, we observed no difference in latency to attack between genotypes (*t* = 0.41*, P* = 0.69) (Figure 2F). Similarly, of the homozygous Ala56 animals, 13/18 showed aggression toward the intruder during the 10-minute test period, as compared to 10/18 homozygous Gly56 animals (Fisher’s exact test *P* = 0.49).

In our prior study on the 129 background, we found decreased ultrasonic vocalizations (USVs) in homozygous Ala56 pups when separated from the dam at postnatal day 7. In contrast, on the B6 background, homozygous Ala56 pups displayed increased USVs in comparison to wildtype littermate controls (Mann–Whitney *U* = 368.5, *P* = 0.017) (Figure 2G). Secondary Kruskal-Wallis comparison of all three genotypes showed a trend for an overall genotype effect (*K-W* 5.59, *P* = 0.06).

**Figure 3:**
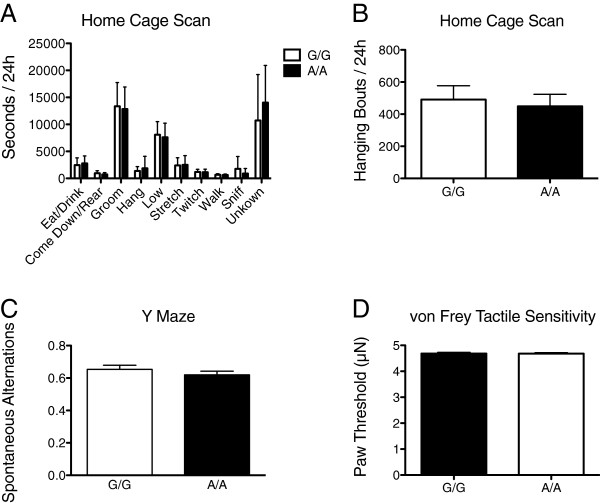
**Home cage, exploratory, and sensory behavior in male B6 SERT Ala56 knock-in mice. A)** Time (mean, standard error of the mean) spent performing individual non-sleep behaviors over 24 hours in the home cage in wildtype littermate controls (G/G, n = 12) and homozygous (A/A, n = 12) SERT Ala56 mice; **B)** Number of bouts of hanging behavior in 24 hours in the home cage; **C)** Ratio of spontaneous alternations, defined as entering a different arm than on the previous two entries, divided by total entries in the Y maze (n = 20 per genotype); **D)** Force threshold at which the paw is moved upon bending of the von Frey filaments (n = 20 per genotype).

No changes in repetitive or sensory behavior were induced by the Ala56 variant on the B6 background. Behavior in the home cage did not show any differences by genotype (Genotype x Behavior Interaction *F* = 0.82, *P* = 0.60) (Figure 3A). There was also no difference between the genotypes when hanging behavior, which was increased on the 129 background, was considered alone (*t* = 0.35*, P* = 0.73) (Figure 3B). Importantly, this behavior was less prevalent on the B6 background in general in comparison to the 129 background [23]. Further, no differences were observed in spontaneous alternations on the Y maze (*t* = 1.20, *P* = 0.25) (Figure 3C) nor in tactile sensitivity assessed using reaction to von Frey filaments (*t* = 0.07*, P* = 0.95) (Figure 3D).

**Figure 4:**
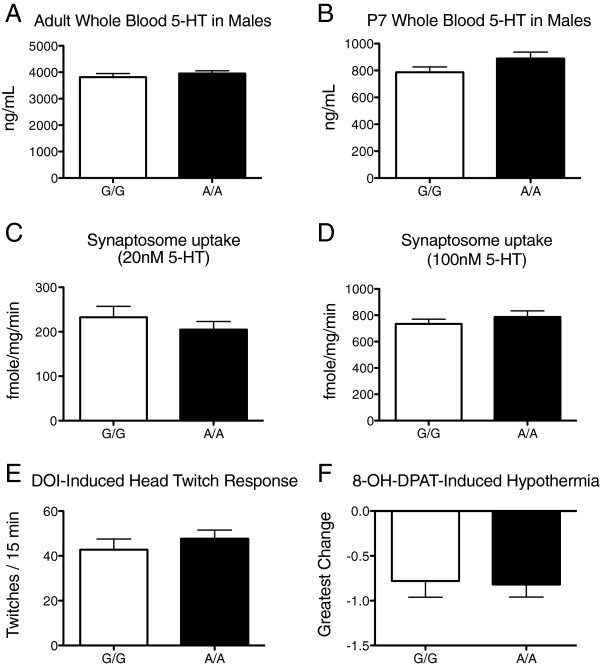
**Assays of the serotonin system in B6 SERT Ala56 knock-in mice. A)** Whole blood serotonin levels (mean, standard error of the mean) in adult male wildtype littermate control (G/G, n = 6) and homozygous SERT Ala56 (A/A, n = 6) mice; **B)** Whole blood serotonin levels in male pups at postnatal day 7 (n = 12 per genotype); **C)** Synaptosome uptake of 20 nM ^3H^5-HT during a 5-minute assay at 37°C, (n = 6 per genotype, with 3 replicates for each subject); **D)** Synaptosome uptake of 100 nM ^3H^5-HT during a 5-minute assay at 37°C (n = 6 per genotype, with 3 replicates for each subject); **E)** Head twitch response to injection of the 5-HT_2_ agonist 2,5-dimethoxy-4-iodoamphetamine (DOI) during a 15-minute observation period (n = 12 per genotype); **F)** Decrease in rectal temperature from baseline in response to injection of the mixed 5-HT_1A/7_ agonist 8-OH-DPAT (n = 10 per genotype).

Since we observed evidence of a genetic background effect on behavioral phenotypes, we examined whether Ala56-induced serotonergic phenotypes were preserved. On the B6 background, we observed no genotype influences on adult male whole blood 5-HT levels (Figure 4A). To decrease variability and maximize sensitivity to detect a differences, we moved to a fluorometric detection method at post-natal day 7 [35] and here observed a trend for higher whole blood 5-HT levels in males, but only when we performed a one-tailed test (*t* = 1.64, P = 0.058, n = 12 per genotype) (Figure 4B). No difference in whole blood 5-HT levels was observed with females (one-tailed *t* = 0.27, P = 0.40, n = 12 per genotype) [See Figure S4 in Additional file 4]. Other studies of serotonergic phenotypes were performed in males only.

*Ex vivo* and *in vivo* measures of brain 5-HT phenotypes also suggested an attenuated impact of the Ala56 variant on a B6 background. Midbrain synaptosome 5-HT uptake showed no significant difference between genotypes at either 20 nM (t = 0.11, *P* = 0.93) or 100 nM of 5-HT (*t* = 2.93, *P* = 0.10) (Figure 4C,D). Since *ex vivo* synaptosome studies may not reproduce changes in 5-HT systems *in vivo*, we also assessed the impact of intraperitoneal (i.p.) injections of 5-HT receptor agonists [23]. In contrast to our prior findings, injection of the 5-HT_2A/2C_ agonist 2,5-dimethoxy-4-iodoamphetamine (DOI) induced a similar number of head twitches in Ala56 and Gly56 animals (*t* = 0.79, *P* = 0.44) (Figure 4E). Similarly, no significant difference was observed between the genotypes in the maximum hypothermia response to injection of the 5-HT_1A/7_ agonist 8-hydroxy-2-(di-n-propylamino)tetralin (8-OH-DPAT) (*t* = 0.16, *P* = 0.87) (Figure 4F).

## Discussion

Strain comparisons have been profitably used to identify quantitative trait loci (QTLs) and specific genes that modify traits, as for example with the recombinant inbred (RI) mouse reference panel derived from C57BL/6 and DBA2/J parental strains [24,36]. In this regard, we recently identified functional coding variation in SERT that distinguishes these two lines (SERT variation is the same in 129 strains as in DBA2/J) and were able to identify multiple anatomical, biochemical and behavioral traits linked to this variation [24]. Strain-dependent genetic variation provides a significant opportunity to identify genetic background effects on the impact of risk alleles, including the SERT Ala56 variant.

Biochemical and behavioral assays displayed a striking divergence of phenotypes between SERT Ala56 knock-in mice on the 129 versus on the B6 genetic background. Whereas a few tests showed quantitative differences in 5-HT related biochemical measures on the B6 background in the same direction as seen for the SERT Ala56 allele on the 129 background, none of these assays achieved statistical significance. We suspect that the complexity of 5-HT homeostasis and signaling [37,38], allows variation in multiple different proteins to moderate the impact of the SERT Ala56 allele. Importantly, we previously showed that the native 129 SERT (Glu39) has higher 5-HT uptake than the Gly39 variant found in the B6 SERT [24]. It is conceivable that the effect of moving the Glu39 SERT onto the B6 background is large enough that additional effects of the Ala56 variant are not detectable. Further studies directly comparing Glu39 and Gly39 SERTs on a B6 background could shed light on this possibility.

Our findings of genetic background influences on behavioral measures are consistent with strain-dependent behavioral phenotypes observed in mouse models of other ASD-associated genes, most prominently including the Fragile X syndrome model *Fmr1* null mouse, which shows variable phenotypes across multiple inbred strains and hybrid crosses [39]. The *NLGN3* R451C knock-in mouse also shows differences in behavior between mixed 129S/B6 and pure B6 inbred strains [40-42]. Further, the SERT null mouse also shows variable behavioral phenotypes across inbred strain backgrounds [43].

The one behavioral phenotype that was consistent across both genetic backgrounds was the tendency for SERT Ala56 mice to back away from mice of other genotypes in the tube test. Not only did the homozygous mutants back out when confronted with wildtype animals, they also backed away when confronted with heterozygous animals. No difference was seen when heterozygous animals and wildtype animals were paired, suggesting that the Ala56 allele only has a significant effect on this phenotype in the homozygous state. From published data, the tube test appears to be a very sensitive test of altered social function in genetic mouse models related to ASD, with significant findings in the Fragile X syndrome model [44], the Rett syndrome model [45], the *Arx*((GCG)10+7) infantile spasms model [46], the Potocki-Lupski model Dp(11)/17/+ mice [47], and the *Dhcr7* heterozygous model of Smith-Lemli-Opitz Syndrome [48]. On the other hand, the tube test may also reveal significant differences in mice with altered motor function or impulsivity, or under conditions of social stress [49], and was originally developed as a test of social hierarchy/dominance, making it a sensitive but not specific test for ASD-relevant social behavior. Social interaction in the tube test does not generate aggression and usually includes only whisker contact. Dominance in this task sometimes [50-52] but not always [53] predicts territorial aggression, and we did not observe any genotype differences in aggression on the resident-intruder test. We isolated resident mice for 7 days, and it is possible that a longer period of social isolation may have generated more aggression and a potential genotype difference. It seems more likely, however, that the tube test phenotype reflects some genotype difference in social judgment that is not simply a difference in territoriality or aggression.

Ultrasonic vocalizations on postnatal day 7 also showed a significant difference between genotypes on the B6 background, but this difference was in the opposite direction of that observed on the 129 background. Importantly, B6 animals are well-known to have age-related hearing loss beginning around 2 to 3 months of age [54], particularly at higher frequencies, so it is possible that the maternal response to ultrasonic vocalizations would be altered on this genetic background. While maternal response could theoretically interact with pup genotype effects on social communication, it remains difficult to reconcile these opposite findings in the SERT Ala56 mice. Of note, some children with ASD are described as having been passive infants, with less crying, whereas others are described as more dysregulated, with more distress and crying [55,56].

Other subtle but significant differences were also found in the SERT Ala56 mice on the B6 background. Mixed results in anxiety-like behavior contrast with decreased anxiety-like behavior in the human SERT over-expressing mouse (hSERT OE) [57,58]. The attenuation of serotonergic phenotypes in the B6 SERT Ala56 mice may account for the difference in findings from reports in the hSERT OE mouse. Alternatively, these discrepant findings could be due to the difference in mechanism between increased (hSERT OE) and dysregulated activity (SERT Ala56 knock-in) [20,21,23].

The male SERT Ala56 mice also show a subtle but significant decrease in activity in the open field, which is actually convergent with findings in the hSERT OE mice, but also in the SERT null mice [43,58,59]. They do not show changes in locomotion in the home cage or in the Y maze, however, suggesting that this could also be a chance finding. A number of potential explanations could explain convergence in activity measures with increased or ablated SERT activity. Both SERT Ala56 and SERT null mice show a decrease in 5-HT neuron firing rate [23,60,61]. Further, Jennings and colleagues showed a loss of frequency sensitivity for evoked 5-HT release in both hSERT OE and SERT null mice, suggesting diminished information processing as a convergent phenotype for opposite disruptions of 5-HT homeostasis [62]. Multiple examples of convergent phenotypes with increased or decreased gene dosage or function have been described in ASD human genetic studies, including *MECP2* deletion and duplication [63-65], chromosome 16p11 deletion and duplication [66], and *SHANK3* deletion and duplication [67].

## Conclusions

In summary, the differences between serotonergic phenotypes on the 129 and B6 backgrounds suggest the presence of modifier loci that impact phenotypic expression of SERT Ala56 in mice, consistent with human genetic data. A cross between these two inbred strain backgrounds (F2 cross) could be used to identify gene variants that modify SERT Ala56-dependent phenotypes and could then be evaluated for epistatic effects with *SLC6A4* variants in human genetic studies. Significant changes in the tube test, despite little difference in many other behaviors or in serotonergic assays in adult animals, suggests that this test of social behavior may be more dependent on SERT activity or regulation. Possibly, this behavior reflects an impact of SERT functional alterations during development that is insensitive to later modifiers that obscure genotype influences on 5-HT traits in adults. Further studies that explore the impact of the SERT Ala56 variant during development are needed to more clearly identify temporal epochs through which SERT and 5-HT signaling impact behavior, as for example with conditional strategies that institute or limit expression of the variant at different stages in the life of the animal.

## Abbreviations

Ala: Alanine; Arg: Arginine; ASD: Autism spectrum disorder; B6: C57BL/6; DOI: 2,5-dimethoxy-4-iodoamphetamine; Glu: Glutamate; Gly: Glycine; HPLC: High-performance liquid chromatography; hSERT OE: Human serotonin transporter over-expressing; Lys: Lysine; SERT: Serotonin transporter; 5-HT1A: Serotonin 5-HT1A receptor; 5-HT2A: Serotonin 5-HT2A receptor; 129: 129S6/S4; 5-HT: 5-hydroxytryptamine (or serotonin); 8-OH-DPAT: 8-hydroxy-2-(di-n-propylamino)tetralin.

## Competing interests

The authors have no direct competing financial interests. JV receives research funding for non-overlapping work from Seaside Therapeutics, Roche Pharmaceuticals, Novartis, Forest, and SynapDx. He has served on an Advisory Board for Novartis and Roche Pharmaceuticals. RDB receives research support for non-overlapping research from Amgen and is a member of the Lundbeck Psychopharmacology Scientific Advisory Board.

## Authors’ contributions

TK participated in study design, data collection, data analysis, and manuscript preparation. CM participated in study design, data collection, data analysis, and manuscript preparation. MM participated in data collection and analysis. CJ participated in data collection. RP participated in data collection. CS participated in data collection and analysis. NB participated in data collection and analysis. GA participated in data collection and analysis. JC assisted in study design and manuscript preparation. JS assisted in study design. RB participated in study design. JV participated in study design, data analysis, and manuscript preparation. All authors read and approved the final manuscript.

## Supplementary Material

Additional file 1: Figure S1 Additional activity, anxiety-like, and social behavior measures in male B6 SERT Ala56 knock-in mice. A) Distance traveled (mean, standard error of the mean) in the first 5 minutes in the open field in wildtype littermate controls (G/G, n = 20), heterozygous (A/G, n = 16), and homozygous (A/A, n = 20) SERT Ala56 knock-in mice; B) Number of entries to the open zone of the elevated zero maze; C) Number of total entries to either the open or the closed zone of the elevated zero maze; D) Time spent in the familiar social stimulus chamber (familiar mouse) or novel social stimulus chamber (novel mouse) in the three-chamber preference for social novelty test for wildtype littermate controls (G/G, n = 20), heterozygous (A/G, n = 16), and homozygous (A/A, n = 20) SERT Ala56 mice; E) Time spent within 1 cm of the familiar or novel social stimulus in the three-chamber preference for social novelty test. * *P* < 0.05. Click here for file

Additional file 2: Figure S2 Activity and anxiety-like behavior in female B6 SERT Ala56 knock-in mice. A) Time (mean, standard error of the mean) spent ambulating during the first 5 minutes in the open field for wildtype littermate controls (G/G, n = 20), heterozygous (A/G, n = 20), and homozygous (A/A, n = 20) SERT Ala56 mice; B) Time spent in the center (>1 cm away from the side) of the open field divided by total time in the open field; C) Time spent in the open zone divided by total time in the elevated zero maze; D) Time spent in the light chamber divided by total time in the light–dark test. Click here for file

Additional file 3: Figure S3 Activity and anxiety-like behavior in female B6 SERT Ala56 knock-in mice. A) Distance traveled (mean, standard error of the mean) in the first 5 minutes in the open field in wildtype littermate controls (G/G, n = 20), heterozygous (A/G), and homozygous (A/A) SERT Ala56 knock-in mice; B) Number of entries to the open zone of the elevated zero maze; C) Number of total entries to either the open or the closed zone of the elevated zero maze. Click here for file

Additional file 4: Figure S4 Whole blood serotonin levels in P7 female B6 SERT Ala56 knock-in mice. Whole blood serotonin levels (mean, standard error of the mean) in female wildtype littermate control (G/G, n = 12) and homozygous SERT Ala56 (A/A, n = 12) pups at postnatal day 7. Click here for file
