# Supplementary figures and images for: Genetic background modulates phenotypes of serotonin transporter Ala56 knock-in mice
Source: Mol Autism. 2013 Oct 1;4:35. doi: 10.1186/2040-2392-4-35 (PMC3851031; doi:10.1186/2040-2392-4-35)

**A**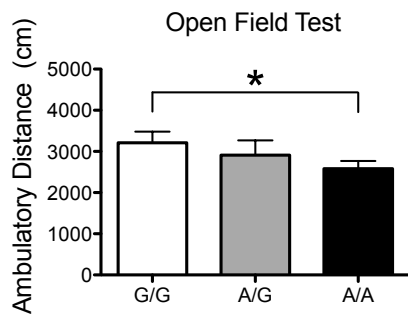**B**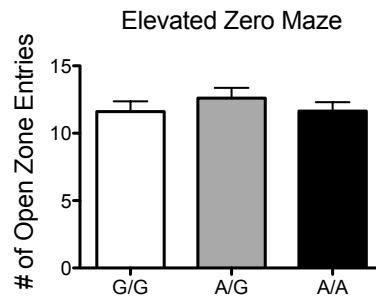**C**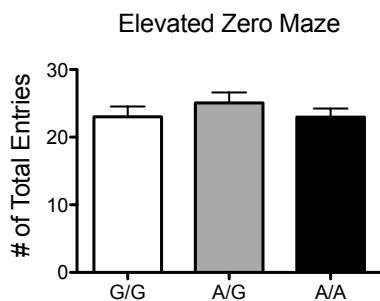**D**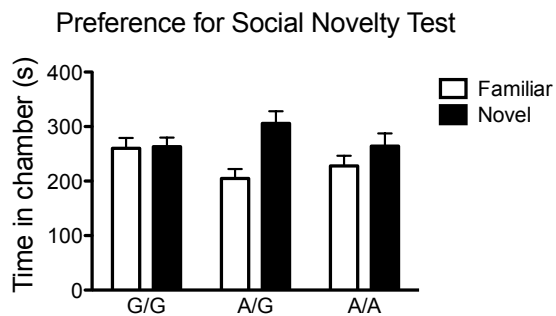**E**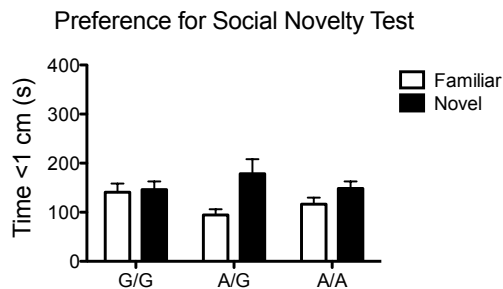

Supplement: Additional file 1: Figure S1 — Additional activity, anxiety-like, and social behavior measures in male B6 SERT Ala56 knock-in mice. A) Distance traveled (mean, standard error of the mean) in the first 5 minutes in the open field in wildtype littermate controls (G/G, n = 20), heterozygous (A/G, n = 16), and homozygous (A/A, n = 20) SERT Ala56 knock-in mice; B) Number of entries to the open zone of the elevated zero maze; C) Number of total entries to either the open or the closed zone of the elevated zero maze; D) Time spent in the familiar social stimulus chamber (familiar mouse) or novel social stimulus chamber (novel mouse) in the three-chamber preference for social novelty test for wildtype littermate controls (G/G, n = 20), heterozygous (A/G, n = 16), and homozygous (A/A, n = 20) SERT Ala56 mice; E) Time spent within 1 cm of the familiar or novel social stimulus in the three-chamber preference for social novelty test. * P < 0.05. [file 2040-2392-4-35-S1.pdf]

**A**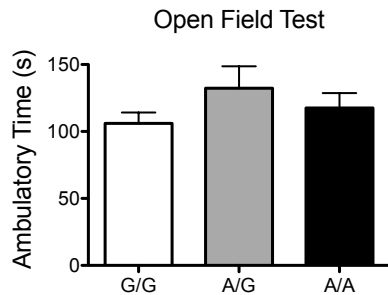**B**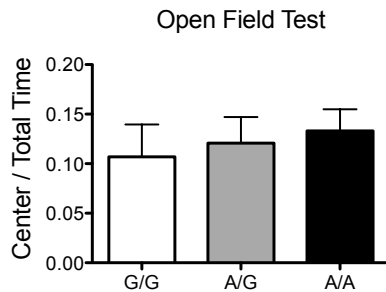**C**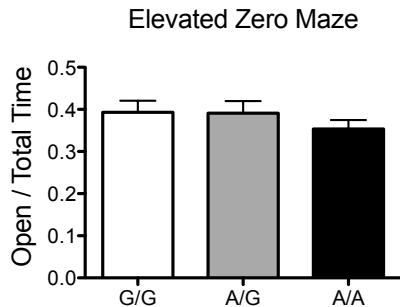**D**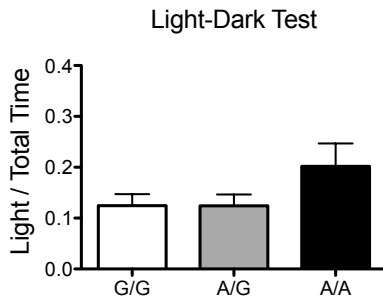

Supplement: Additional file 2: Figure S2 — Activity and anxiety-like behavior in female B6 SERT Ala56 knock-in mice. A) Time (mean, standard error of the mean) spent ambulating during the first 5 minutes in the open field for wildtype littermate controls (G/G, n = 20), heterozygous (A/G, n = 20), and homozygous (A/A, n = 20) SERT Ala56 mice; B) Time spent in the center (>1 cm away from the side) of the open field divided by total time in the open field; C) Time spent in the open zone divided by total time in the elevated zero maze; D) Time spent in the light chamber divided by total time in the light–dark test. [file 2040-2392-4-35-S2.pdf]

**A**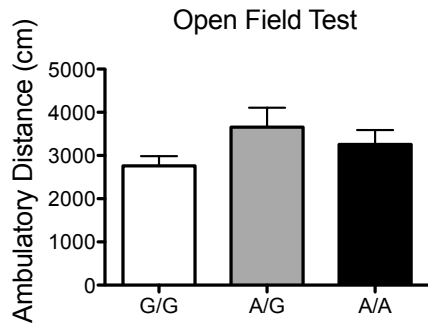**B**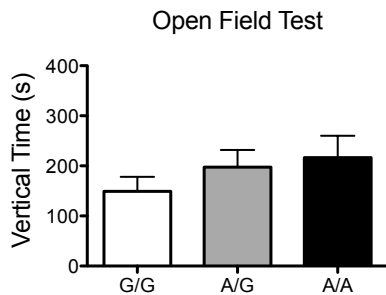**C**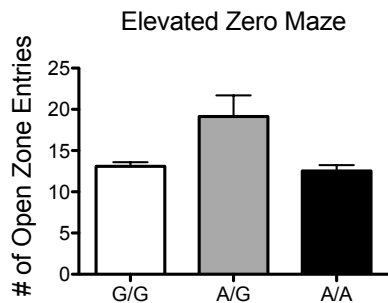**D**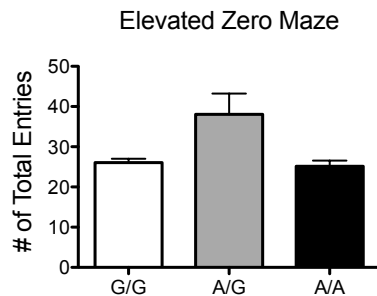

Supplement: Additional file 3: Figure S3 — Activity and anxiety-like behavior in female B6 SERT Ala56 knock-in mice. A) Distance traveled (mean, standard error of the mean) in the first 5 minutes in the open field in wildtype littermate controls (G/G, n = 20), heterozygous (A/G), and homozygous (A/A) SERT Ala56 knock-in mice; B) Number of entries to the open zone of the elevated zero maze; C) Number of total entries to either the open or the closed zone of the elevated zero maze. [file 2040-2392-4-35-S3.pdf]

## P7 Whole Blood 5-HT in Females

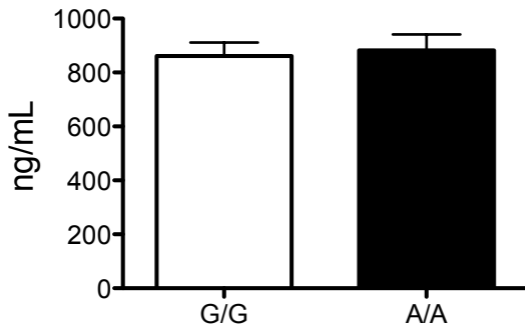

Supplement: Additional file 4: Figure S4 — Whole blood serotonin levels in P7 female B6 SERT Ala56 knock-in mice. Whole blood serotonin levels (mean, standard error of the mean) in female wildtype littermate control (G/G, n = 12) and homozygous SERT Ala56 (A/A, n = 12) pups at postnatal day 7. [file 2040-2392-4-35-S4.pdf]
